# Recovery from apoptosis in photoreceptor cells: A role for mitophagy

**DOI:** 10.1038/s41419-026-08436-3

**Published:** 2026-01-30

**Authors:** Bhavneet Kaur, Bruna Miglioranza Scavuzzi, Jingyu Yao, Mengling Yang, Lin Jia, Stephen I. Lentz, Jaya Sadda, Andrew J. Kocab, Sumathi Shanmugam, David N. Zacks

**Affiliations:** 1 Department of Ophthalmology and Visual Sciences, Michigan Medicine, Kellogg Eye Center, University of Michigan, Ann Arbor, MI 48105 USA; 2 Department of Ophthalmology, Xiangya Hospital, Xiangya School of Medicine, Central South University, Changsha, Hunan China; 3 ONL Therapeutics, Inc, Ann Arbor, MI 48104 USA

**Keywords:** Apoptosis, Mitophagy

## Abstract

Photoreceptors (PRs) are specialized light-sensitive cells responsible for vision, and their death is the primary cause of retinal degeneration and vision loss. Recent studies using cells such as HeLa and PC12 have demonstrated cellular recovery even from late stages of apoptosis. Here, we demonstrate for the first time that PR cells can recover from features of apoptosis following exposure to apoptotic stressors. Upon apoptotic stimuli (staurosporine or hypoxia), 661 W cells, a murine cone PR cell line, exhibited morphological and functional features of apoptosis, such as rounding and blebbing, caspase-3 activation, PARP cleavage, and phosphatidylserine externalization. These processes were reversed upon the alleviation of stress. We also observed that mitochondrial function is central to apoptotic recovery of photoreceptor cells, as evidenced by the restoration of intracellular ATP levels and reduction in mitochondrial reactive oxygen species (mROS). Mitophagy was demonstrated to play a crucial role in cell survival, with increased protein and mRNA expression of mitophagy markers during recovery from apoptosis. Furthermore, the modulation of mitophagy confirmed its protective role in the recovery phase, as its induction with MF-094 reduced apoptosis while its inhibition with Mdivi-1 exacerbated cell death. In vivo, we demonstrate the recovery of PRs from apoptosis using an experimental model of transient retinal detachment. Altogether, the findings of this study indicate that PR cells can recover from entry into the apoptotic cascade, and that mitophagy is essential for apoptotic recovery in these cells.

## Introduction

Photoreceptors (PRs) are specialized cells responsible for converting light into neural signals [1]. Their inability to proliferate makes them vulnerable to damage and once they undergo apoptosis, they cannot be replaced, leading to permanent vision loss [2]. PR degeneration is a hallmark of numerous retinal diseases, including retinitis pigmentosa, age-related macular degeneration (AMD), and retinal detachment (RD) [2–5]. RD, defined as the separation of the retina from the underlying retinal pigment epithelium (RPE), triggers numerous molecular and structural changes to the PRs, including the shedding and shortening of outer segments [6–8], changes in protein synthesis patterns [9, 10], and activation of both pro-survival and pro-death pathways [11–16]. Apoptosis and necroptosis have been implicated in PR cell death; however, it is well established that apoptosis is the predominant cause of PR death after RD, and other retinal diseases [13, 17–19]. Because the loss PR cells often leads to irreversible vision loss, mechanisms that allow survival after stress are particularly valuable. If retinal cell recovery allows a stressed or apoptotic retinal cell to recover, then vision might be preserved rather than lost. It has been shown that retinal ganglion cells (RGCs) can recover from early stages of cell death activation if the death stimulus is removed before mitochondrial cytochrome c release and plasma membrane phosphatidylserine exposure. Specifically, they discovered that RGCs with mitochondrial fragmentation and membrane potential loss could reverse damage [20]. In this context, finding novel strategies to reduce or reverse cellular apoptosis after its initiation is of critical importance.

Derived from the Greek word for “rising to live”, anastasis is the ability of cells to reverse the apoptotic process and recover after the removal of an apoptotic stimulus [21]. The concept of anastasis challenges the long-standing belief that once activated, the apoptotic cascade is irreversible [22]. Traditionally, apoptosis was viewed as a tightly regulated, unidirectional process essential for maintaining tissue homeostasis and eliminating damaged or unnecessary cells [23–25]. However, recent evidence in various cell types has revealed that cells can survive apoptotic stimulus and regain normal function once the apoptotic stimulus is removed [22, 26–28]. Thus, unlike cellular recovery from sublethal stimuli, anastasis is a distinct process defined as the recovery of cells after they have initiated and even executed key steps of apoptosis which have historically been considered “points of no return”, such as membrane blebbing, caspase activation and PARP cleavage [22, 29].

Mitochondrial homeostasis plays a central role in apoptosis and cellular recovery during recovery from apoptosis [30]. During apoptotic stress, mitochondrial dysfunction leads to ATP depletion, increased reactive oxygen species (ROS) production, and cytochrome c release, ultimately activating caspases [31–33]. If apoptotic stimuli are removed during the occurrence of apoptosis, mitochondrial function may be restored, allowing cell survival [34]. Mitochondrial fusion and fission are tightly linked to the cellular response to mitochondrial apoptosis [35]. Fusion supports recovery by restoring mitochondrial function, while fission plays a key role in promoting apoptosis when recovery is not possible [35]. Together, these processes maintain mitochondrial integrity, help manage stress and determine whether a cell survives or undergoes programmed cell death.

One key process that facilitates mitochondrial recovery is mitophagy, a selective form of autophagy that eliminates damaged mitochondria and promotes mitochondrial biogenesis [36]. Previous studies have identified mitophagy regulators such as Pink1, Fundc1, and Parkin as essential for cellular survival under stress conditions [36]. However, the role of mitophagy in recovery from apoptosis, particularly in PR, remained largely unexplored. In this study, we hypothesized that PR cells can also undergo recovery from apoptotic insult. To test this hypothesis, we exposed 661 W cells, a murine cone PR cell line, to two different apoptotic stress stimuli, namely staurosporine (STR), which is considered a reference agent for the induction of apoptosis via the intrinsic pathway [37] and hypoxia (HYP), which is a driver of apoptotic cell death in the retina in conditions where there is a separation of the retinal pigmented epithelium and the neuronal retina, such as RD and AMD. In addition, we established a methodology for inducing transient retinal detachment (tRD) in mice using a 0.033% sodium hyaluronate solution. This approach produces a detachment similar to those described in previous models, but with natural reattachment occurring within 3 days. This mimics the reattachment observed in vivo following surgical intervention and effectively models the resolution of hypoxic stress, providing a relevant in vivo system for studying recovery from apoptosis. Both in vitro and in vivo, we observed evidence of cellular recovery from apoptotic insults, thereby preventing irreversible cell death. Understanding the mechanisms that govern recovery from apoptotic insults could provide novel therapeutic strategies for preserving photoreceptors in degenerative conditions.

## Material and methods

All materials utilized in this study, including reagents, equipment, vendor names and catalog numbers can be found in Supplemental Table 1.

### Cell Culture

The photoreceptor cell line 661 W, was kindly donated by Dr. Muayyad Al-Ubaidi (University of Houston, Houston, TX), and was used for the in vitro evaluations of the study [38]. Cells culture conditions and media have been previously detailed by our group [39]. In short, media consisted of Dulbecco’s Modified Eagle Medium (DMEM) supplemented with 10% fetal bovine serum, 1% penicillin–streptomycin, 32 mM putrescine, 40 µL/L β-mercaptoethanol, 40 µM hydrocortisone 21-hemisuccinate, and 40 µM progesterone.

### Models of apoptosis induction

**Fig. 1:**
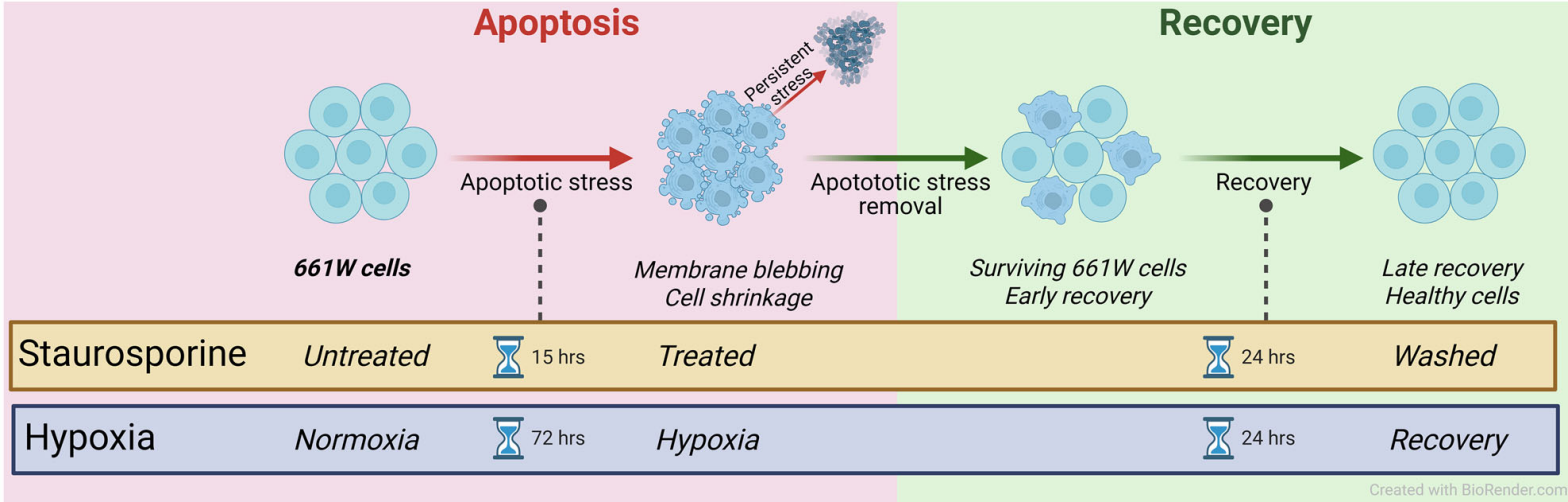
Experimental protocols to evaluate recovery from apoptosis in 661 W cells. 661 W cells were subjected to apoptotic stress using two different protocols: staurosporine treatment (15 h) or hypoxia (72 h). Both stressors induce apoptosis, characterized by morphological changes, phosphatidylserine externalization, and caspase-3 activation. If stress is persistent, cells exhibit membrane blebbing and apoptotic body formation. However, upon removal of the stressor, cells demonstrate recovery of apoptotic morphology and functional features, indicating reversal of apoptosis. Each protocol includes three experimental groups: Staurosporine protocol – group receiving no staurosporine treatment (Untreated), group receiving 15-h 0.05 µM staurosporine treatment (Treated), and group receiving 15-h 0.05 µM staurosporine followed by 24-h recovery (Washed). Hypoxia protocol – group in normoxic conditions (Normoxia), group receiving 72-h hypoxic treatment (Hypoxia), and group receiving 72-h hypoxic treatment followed by 24-h of recovery (Recovery).

To evaluate cellular recovery by recovery from apoptotic insults in vitro, 661 W cells were subjected to apoptotic stress using two different protocols: either a 15-h 0.05 µM staurosporine treatment, or a 72-h hypoxic treatment, as shown in Fig. 1. For hypoxic treatment, cells were subjected to hypoxic conditions for 72 h using a specialized incubator chamber, filled with humidified hypoxic air (1% O_2_, 5% CO_2_, 94% N_2_), in a methodology previously detailed by our group [40]. After the appropriate treatment times, cell culture media was replaced, and cells were allowed to recover for 24 h in both protocols.

Each protocol included three experimental groups. For the Staurosporine protocol, there was a group receiving no staurosporine treatment (Untreated), a group receiving 15-h 0.05 µM staurosporine treatment (Treated), and a group receiving 15-h 0.05 µM staurosporine followed by 24-h recovery after the renewal of cell culture media (Washed). For the Hypoxia protocol, there was a group in normoxic conditions (Normoxia), a group receiving 72-h hypoxic treatment (Hypoxia), and a group receiving 72-h hypoxic treatment followed by 24-h of recovery under normoxic conditions after the renewal of cell culture media (Recovery). Experimental protocol is depicted in Fig. 1. A total of 72 h of hypoxia exposure was selected based on our previous finding that this time period was required for approximately 40% of cell death [39].

### Permanent and Transient Retinal Detachment mouse models

Adult (male/female) C57BL6J mice of 8 to 10 weeks of age procured from The Jackson Laboratory were used for this study. Housing and anesthetizing conditions have been previously detailed by our group [11]. In this study, both permanent RD and tRDs were performed. In short, a sclerotomy was created approximately 1 mm posterior to the limbus using a 30-gauge needle. Subsequently, 1% sodium hyaluronate (Healon®PRO) was carefully injected into the subretinal space with a 34-gauge needle until approximately 50% of the retina became detached from the RPE (permanent detachment protocol). For transient detachment of half of the retina, a 3 μL of a 0.033% sodium hyaluronate solution was used under the same conditions, see Supplemental Material for detailed transient detachment protocol. The eyes were enucleated 72 h (3 dprd) or 168 h (7 dprd) after RD in both protocols.

### Western blot analysis

Protein was isolated from retinas as previously detailed [41]. Cells and retinal tissues were lysed using radioimmunoprecipitation assay (RIPA) buffer with phosphatase and protease inhibitors. Protein concentrations were measured using the RC DC™ Protein Assay Kit, according to manufacturer’s protocol. After denaturation, 20 µg of protein was separated and transferred to a polyvinylidene fluoride (PVDF) membrane. Membranes were blocked using a 5% bovine serum albumin in a tris-buffered saline buffer solution (TBS), incubated overnight with the primary antibodies, washed three times with a Tris-buffered saline with 0.1% Tween 20 solution (0.1%TBS-T), and then incubated for 2 h with secondary antibodies, followed by three other rounds of 10-min washes using 0.1%TBS-T. Signals were then detected using chemiluminescent western blotting substrate in the Azure c500 Gel Imaging System. Band intensities were determined using ImageJ (version 1.53 m). Antibodies and reagents utilized are listed in Supplemental Table 1. Full length western blots are presented in Supplemental Material. Experiments were performed in three or more biological replicates. Protein band intensities were first normalized to loading controls and subsequently expressed as fold-change relative to the untreated controls.

### RNA Extraction, cDNA Preparation and mRNA expression evaluation

Total RNA was extracted from cell lysates and retinas using the RNeasy Plus Micro kit, according to supplier’s instructions. Complementary DNA (cDNA) was synthesized using the High-Capacity cDNA Reverse Transcription Kit, in accordance with the manufacturer’s protocol. mRNA expressions were evaluated using a total of 10 µL of a solution containing Fast SYBR Green Master Mix using the CFX384 Touch Real-Time PCR System. Gene expression levels were estimated using the ΔΔCt method, using *Pum1* as the endogenous housekeeping gene [42, 43]. Information on reagents and primer pair sequences is provided in Supplemental Tables 1, 2. Experiments were performed in three or more biological replicates, and each assay included 2 technical replicates within each experiment. Individual plots in graphs represent the mean of the technical replicates.

### Crystal violet

The crystal violet assay was performed to measure cellular proliferation, based on a previously described methodology [44–46]. In brief, at the endpoint of the different apoptosis induction protocols, cellular supernatant was discarded, adherent cells were gently washed with PBS, which was then removed by aspiration. The cells were stained for 15 min with a solution containing 0.5% of crystal violet reagent in 20% methanol. After staining, the cell culture wells were rinsed with water three times to remove excess dye. The crystal violet dye was then solubilized in methanol, and absorbance evaluated at 595 nm. Experiments were performed in three or more biological replicates, and each assay included 6 or more technical replicates within each experiment. Individual plots in graphs represent the mean of the technical replicates.

### Mitochondrial functional assays

*MTT Assay:* Concentrations of inducers and inhibitors used in this study were determined based on results of cellular viability/cytotoxicity employing the 3-(4,5-dimethylthiazol-2-yl)-2,5-diphenyltetrazolium bromide (MTT) methodology [47]. Briefly, at the end of the different apoptosis induction protocols, cellular supernatants were removed, adherent cells were gently washed with PBS, which was subsequently aspirated. Cells were incubated at 37°C for 3 h with a 0.5 mg/mL of Thiazolyl Blue Tetrazolium Bromide reagent in PBS. The resulting dark-blue formazan crystals, retained only by viable cells due to their membrane impermeability, were solubilized in isopropanol, and absorbance values determined at 540 nm.

Additional mitochondrial functional assays, including levels of intracellular adenosine triphosphate (ATP) and evaluation of mitochondrial ROS were performed as previously detailed by our group [39]. Experiments were performed in three or more biological replicates, and each assay included 2 or more technical replicates within each experiment. Individual plots in graphs represent mean of the technical replicates. Results from different biological replicates were expressed as fold-change relative to the untreated controls.

### Live-cell imaging

661 W cells (1 × 10⁵) were seeded in 35 mm glass-bottom dishes and treated with STR for 15 h. Following treatment, cells were gently washed with PBS to remove residual STR and incubated with 5 µM DRAQ5 dye at 37°C for 2 min for nuclear staining. Warm complete cell culture medium was added, and the cells were immediately imaged. Live-cell images were taken at 15-min intervals using a Nikon Inverted TiE Widefield and A1 Confocal Microscope equipped with CO_2_ supply. Images were captured at a 40x Magnification.

### Apoptosis assessment

*RealTime-Glo Annexin V Apoptosis Assay (Promega)*: was used to assess exposure of phosphatidylserine (PS) on the outer leaflet of the cell membrane during the apoptotic process, according to the supplier’s protocol. Experiments were performed in three or more biological replicates, and each assay included 6 or more technical replicates within each experiment. Individual plots in graphs represent mean of the technical replicates.

*Flow cytometry:* 661 W cells (1 × 10⁵) were seeded into 6-well plates and treated following the standard experimental protocol (see Fig. 1). For cell dissociation, Versene solution was used. This solution was prepared by dissolving 0.1 g EDTA disodium salt, 2.0 g sodium chloride, 0.05 g disodium phosphate, and 0.05 g potassium chloride in Milli-Q water. The pH was adjusted to 7.4, and the final volume brought to 250 mL with Milli-Q water. The prepared Versene solution was filtered through a 0.22 µm filter before use. After completion of the experimental treatments, cell supernatants from all groups (Control, STR, and Washed) were collected immediately prior to experimental analysis and transferred into labeled Eppendorf tubes. Each well was rinsed once with cold PBS, and the supernatant was transferred to the corresponding tubes. The remaining attached cells were incubated with Versene (EDTA) for 10 min at 37°C, collected, and transferred to the appropriate tubes. Wells were then rinsed one final time with cold PBS, and these washes were also collected. All samples were centrifuged at 4000 rpm for 5 min at 4°C. The cell pellets were washed with cold PBS and resuspended in 100 µL of 1X Annexin Binding Buffer containing FITC, Propidium Iodide (PI) dyes, incubating for 15 min according to the manufacturer’s protocol (Dead Cell Apoptosis Kits with Annexin V for Flow Cytometry, Invitrogen). Internal controls (unstained, FITC only, and PI only) were prepared using the appropriate dyes and following the same protocol. Cells were analyzed using flow cytometry (Attune), with 10,000 events collected per sample. Cellular status was identified using plot analysis based on staining profiles: healthy/viable cells (Annexin V⁻/PI⁻), early apoptotic cells (Annexin V⁺/PI⁻), late apoptotic cells (Annexin V⁺/PI⁺), and necrotic cells (Annexin V⁻/PI⁺), and results were reported as the percentage of events out of the total number of cells.

*Terminal Deoxynucleotidyl Transferase-Mediated dUTP Nick-End Labeling (TUNEL) Assay and Retinal Histology:* Apoptosis was assessed in retinal cryosections using the DeadEnd™ Fluorometric TUNEL System following supplier’s protocol, as previously detailed by our group [39, 48]. Evaluations were made three [3] and seven [7] days post-detachment (transient and permanent). Images were acquired utilizing the Leica STELLARIS 8 FALCON Confocal Microscope (Leica Microsystems, Wetzlar, Germany). Retinal histology was performed as previously detailed by our group [49]. Retinal histology was assessed using hematoxylin and eosin staining on 6 µm paraffin sections as described previously [43]. Cornea of the eyes was marked at the 12 o’clock for orientation, then fixed in 4% paraformaldehyde overnight at 4°C before paraffin embedding. Paraffin sections (6 µm) were produced using a microtome. Only sections crossing the optic nerve, containing both the nasal and temporal aspects of the retina were used for staining and images were obtained with a Leica DM6000 microscope.

### Statistical analysis

Statistical evaluations were performed using GraphPad Prism version 10.2.2 for Windows. Graphs in figures are presented as the mean value ± standard error of the mean (SEM). Statistical analyses were performed using one-way ANOVA with repeated measures, followed by Tukey’s post hoc test. Significance levels are indicated as * for *p* < 0.05, ** for *p* < 0.01, *** for *p* < 0.001, and **** for *p* < 0.0001, ns: not significant (*p* > 0.05) in figure graphs.

Graphs reflect mean of technical replicates in each biological replicate.

## Results

### 661 W cells regain homeostatic morphology and function after apoptotic stimuli

**Fig. 2:**
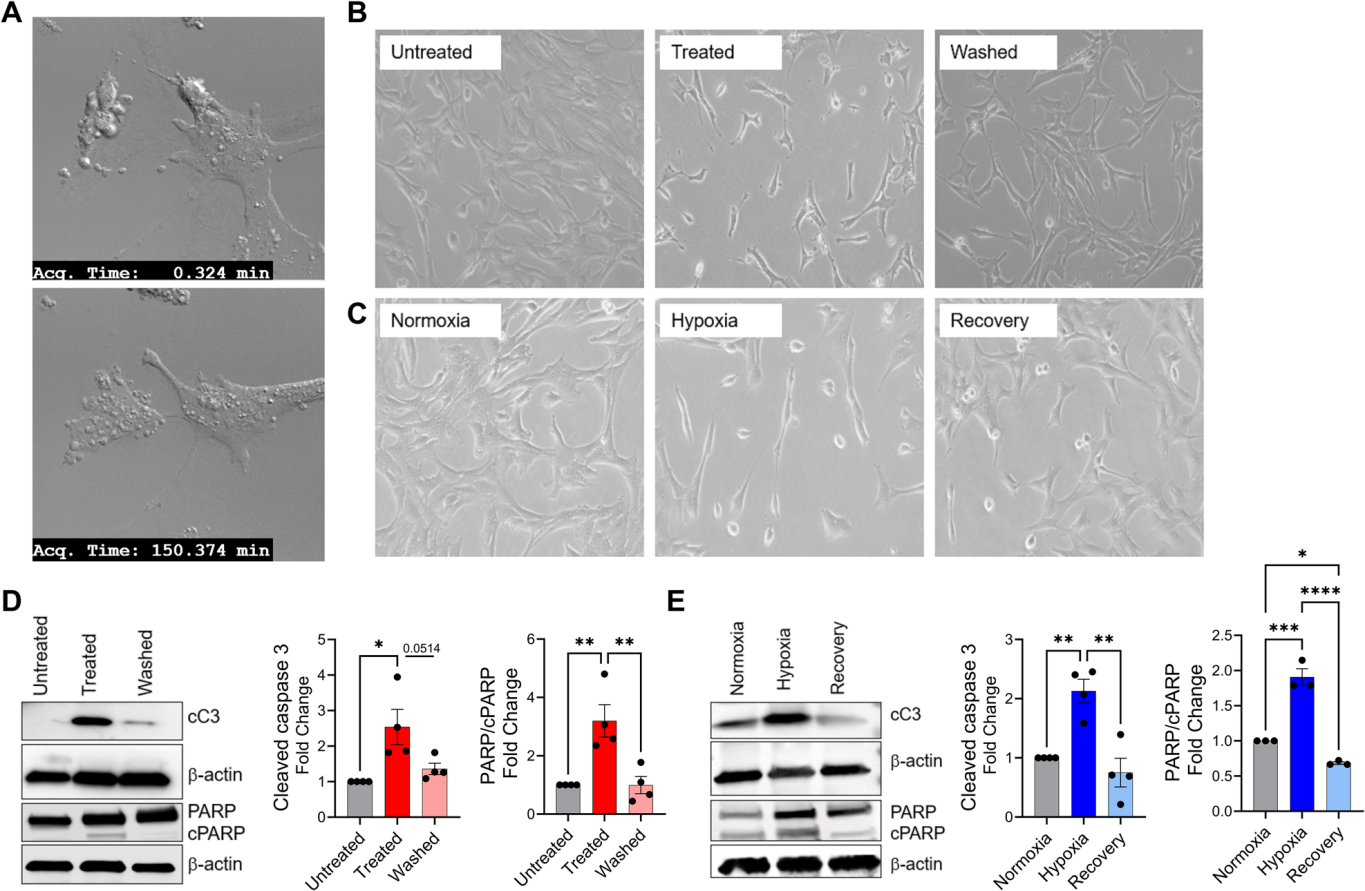
Recovery of morphological and functional features after apoptotic response to staurosporine and hypoxia stress stimuli. **A** Live-cell images captured immediately after staurosporine removal (0.324 min) and following 2.5 h of recovery (150.374 min), illustrating reversal of apoptotic morphological features, including cell rounding and membrane blebbing in the same cells. **B** Representative brightfield images illustrating cellular morphology in the staurosporine protocol: the group without staurosporine treatment (Untreated), the group receiving 15 h of 0.05 µM staurosporine treatment (Treated), and the group receiving 15 h of 0.05 µM staurosporine followed by 24 h of recovery (Washed). **C** Representative brightfield images showing cellular morphology in the Hypoxia protocol: the group in normoxic conditions (Normoxia), the group undergoing 72 h of hypoxic treatment (Hypoxia), and the group receiving 72 h of hypoxic treatment followed by 24 h of recovery in normoxia (Recovery). **D** Representative immunoblots for cleaved caspase 3 (cC3) (*n* = 4), PARP/cleaved PARP (cPARP) (*n* = 4), normalized to β-actin, with respective quantifications for the staurosporine protocol (red). **E** Representative immunoblots for cleaved caspase 3 (cC3) (*n* = 4), PARP/cleaved PARP (cPARP) (*n* = 3), normalized to β-actin, with respective quantifications for the staurosporine protocol (red) and **E** the hypoxia protocol (blue). Bar charts display the average values along with the standard error of the mean (SEM). Statistical analysis was performed using one-way ANOVA with repeated measures, followed by Tukey’s post hoc test. Significance levels are indicated as * for *p* < 0.05, ** for *p* < 0.01, *** for *p* < 0.001, and **** for *p* < 0.0001.

In both the staurosporine (STR) and hypoxia (HYP) protocols, treatment with 15 h of 0.05 µM staurosporine or 72 h of hypoxia, respectively, led to apoptotic morphological features such as cytoplasmic shrinkage, cellular rounding, and blebbing. Cells returned to their homeostatic morphology as early as 2.5 h (Fig. 2A), after the removal of the stress stimuli in both the STR and HYP protocols, regaining complete recovery of homeostatic morphology by 24 h (Fig. 2B, C, respectively). Single-cell tracking of morphological features by live-cell imaging demonstrates a late-apoptotic cell following STR treatment (marked by an arrow) transitioning from a rounded and blebbed appearance, almost detaching from the dish at time- zero, to its characteristic homeostatic elongated morphology by 316 min, other cells in the same frame can be observed recovering homeostatic morphology (Supplementary Video 1).

In addition to morphological changes, staurosporine and hypoxia led to caspase-3 cleavage (*P* = 0.0139 and *P* = 0.0044, respectively) and PARP cleavage (*P* = 0.0051 and P = 0.0002, respectively) in comparison to the control untreated groups. In all cases, 24 h of recovery after the removal of stress stimuli led to a complete restoration of pre-induction levels of cleaved caspase 3 and PARP (Fig. 2D, E). Flow cytometry evaluation indicated that after 24 h of recovery following STR removal, the fraction of Annexin-/PI- (healthy) cells increased from 14% (STR) to 51.7% (Washed). In addition, the proportion of Annexin + /PI- (early apoptosis) cells decreased from 26% (STR) to 17.4% (Washed), and the proportion of Annexin + /PI+ (late apoptosis) cells decreased from 53.7% (STR) to 23.6% (Washed), suggesting recovery to a healthy state even at late stages of apoptosis (Supplemental Fig. 1).

To determine whether 661 W cells reestablished their proliferative capacity after the recovery phase in both protocols, we estimated the number of living cells using the crystal violet assay, which is based on the binding of the crystal violet dye to proteins and DNA, thus allowing an indirectly determination of differences in cellular proliferation after apoptosis induction in the different apoptosis induction protocols [44]. As shown in Supplemental Fig. 2A, B, both treatments significantly reduced cell numbers, as indicated by decreased crystal violet staining in both the STR and HYP protocols (*P* = 0.0001, *P* < 0.0001 respectively). Cellular proliferation was assessed at 24 h in both groups and was observed only in the recovery phase of the HYP protocol (*P* < 0.0001), also indicating cellular recovery. For the hypoxia protocol, at 12 h, although minimum proliferation had occurred ( ~ 11%), levels of apoptosis assessed by Annexin V were significantly lower and levels of cleaved caspase-3 were already virtually undetectable. This suggests that the reduction of levels of markers of apoptosis are not an artifact of cellular proliferation, but rather a reflection of cellular recovery (Supplemental Fig. 2C–E).

### Mitochondrial recovery following apoptotic and hypoxic stress: restoration of ATP, ROS levels and biogenesis markers

**Fig. 3:**
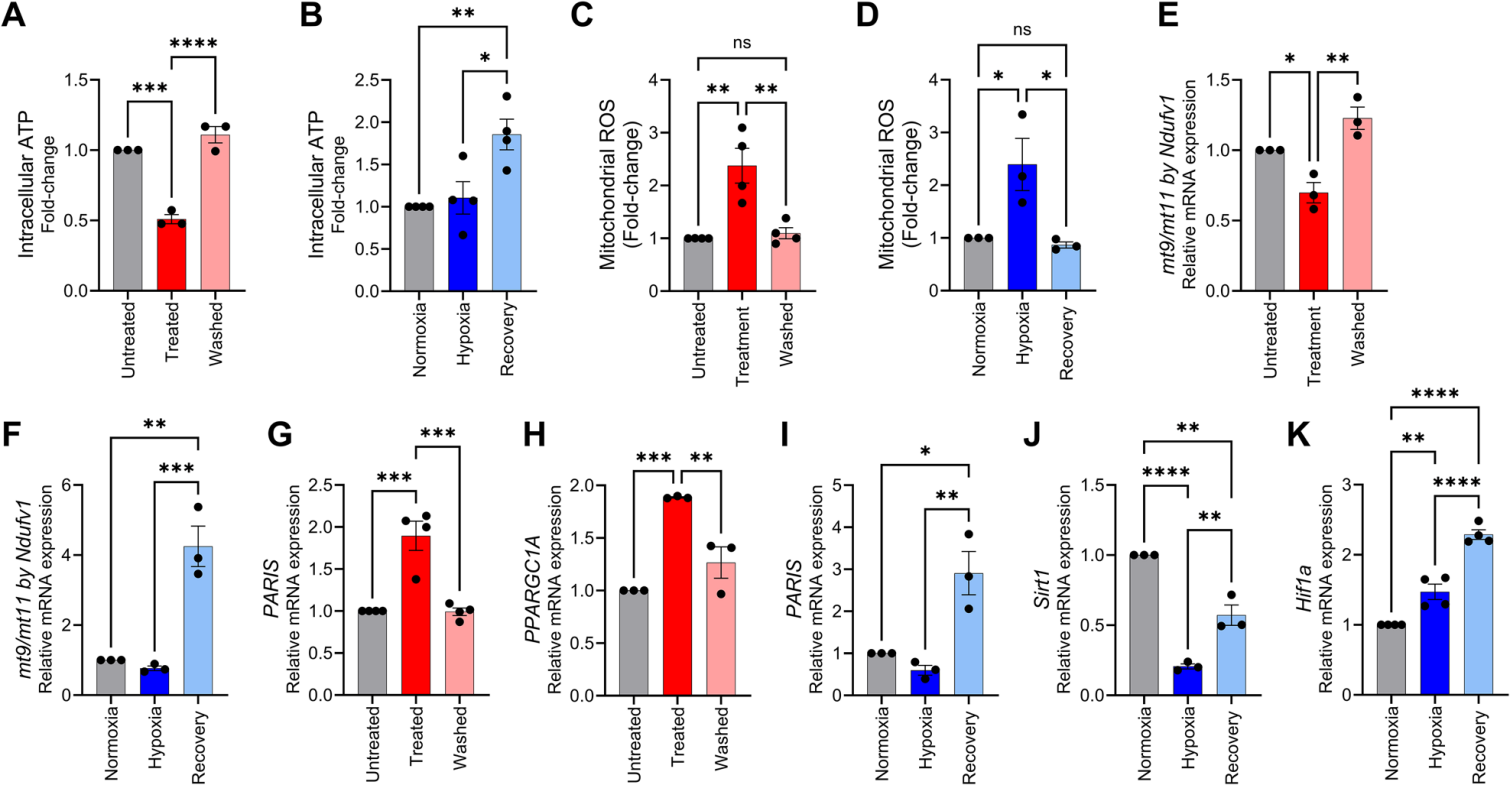
Recovery of mitochondrial function in 661 W cells following apoptotic stimuli induced by staurosporine and hypoxia. Intracellular ATP levels in 661 W cells during **A** staurosporine (*n* = 3) and **B** hypoxia protocols (*n* = 4). Mitochondrial reactive oxygen species (ROS) levels measured using the MitoSOX superoxide indicator in cells subjected to **C** staurosporine (*n* = 4) and **D** hypoxia protocols (*n* = 4). Relative mRNA expression of *mt9/mt11*, normalized to *Ndufv1*, in cells subjected to **E** staurosporine (*n* = 3) and **F** hypoxia (n = 3) protocols. Relative mRNA expression of **G** *PARIS* (*n* = 4) and **H** *PPARGC1A* (*n* = 3) in cells recovering from the staurosporine protocol. Relative mRNA expression of **I** *PARIS* (*n* = 3), **J** *Sirt1* (*n* = 3) and **K** *Hif1a* (n = 4) in cells recovering from the hypoxia protocol. Bar charts display the average values along with the standard error of the mean (SEM). Statistical analysis was performed using one-way ANOVA with repeated measures, followed by Tukey’s post hoc test. Significance levels are indicated as * for *p* < 0.05, ** for *p* < 0.01, *** for *p* < 0.001, and **** for *p* < 0.0001.

Previous studies on recovery from apoptotic insult have identified a central role for the mitochondria in coordinating cellular recovery to apoptosis [27, 30]. Considering that the retina is a tissue with high energy-demands [50], we began assessing mitochondrial function by evaluating intracellular levels of ATP. STR exposure significantly reduced intracellular ATP levels compared to untreated controls (*P* < 0.0001). Upon removal of the apoptotic stimulus, the recovery group exhibited a notable restoration of ATP levels compared to the STR-treated group (*P* = 0.0005), ultimately reaching levels comparable to the untreated control (*P* = 0.2383, Fig. 3A). In contrast, hypoxia (HYP) treatment did not significantly alter intracellular ATP levels relative to normoxic controls (*P* = 0.9299). However, during the recovery phase, ATP levels were significantly elevated in the hypoxia recovery group compared to both the normoxic group (*P* = 0.0065) and the hypoxia-treated group (*P* = 0.0112, Fig. 3B).

Due to the central role of mitochondrial reactive oxygen species (ROS) in regulating cellular apoptosis and recovery from apoptotic insult, we assessed mitochondrial superoxide levels, which were markedly increased by both STR and HYP treatments (*P* < 0.0001 for both). Notably, ROS levels were fully normalized after 24 h of recovery (*P* = 0.0001 for STR; *P* < 0.0001 for HYP, Fig. 3C, D). To further probe mitochondrial function, we examined the ratio of mitochondrial transcripts *mt9* and *mt11*, which can indicate changes in mitochondrial DNA copy numbers, normalized to the nuclear-encoded Complex I subunit *NDUFV1* to account for variations in total DNA content. Following the removal of STR and HYP treatments, this transcript ratio increased significantly compared to their respective treated groups (*P* = 0.0023 for STR; *P* = 0.0008 for HYP), mirroring the observed ATP recovery patterns (Fig. 3F, G).

Mitochondrial biogenesis was assessed by measuring mRNA levels of *Parkin interacting substrate* (*PARIS)*, *Peroxisome proliferator-activated receptor-gamma coactivator-1alpha* (*PPARGC1A*) – which encodes for the PGC-1α protein - and sirtuin 1 gene (*Sirt1*). STR treatment induced a significant increase in mRNA levels of *Paris* and *Pgc1α* (*P* = 0.0005, *P* = 0.0008, respectively), which reestablished baseline values after removal of the apoptotic stimulus (*P* = 0.9984, *P* = 0.1534, respectively), (Fig. 3 H,I). For the hypoxia protocol, mRNA levels of *Paris* were not increased by hypoxia treatment in relation to the normoxic control. Interestingly, *Paris* expression remained unaffected by hypoxia but was significantly increased during the recovery phase (*P* = 0.0041, Fig. 3J). Hypoxia treatment caused a pronounced decrease in *Sirt1* mRNA levels (*P* < 0.0001), which were significantly restored during recovery (*P* = 0.0024, Fig. 3K).

### Regulation of mitochondrial fission and fusion in 661 W cellular recovery from apoptotic insult

**Fig. 4:**
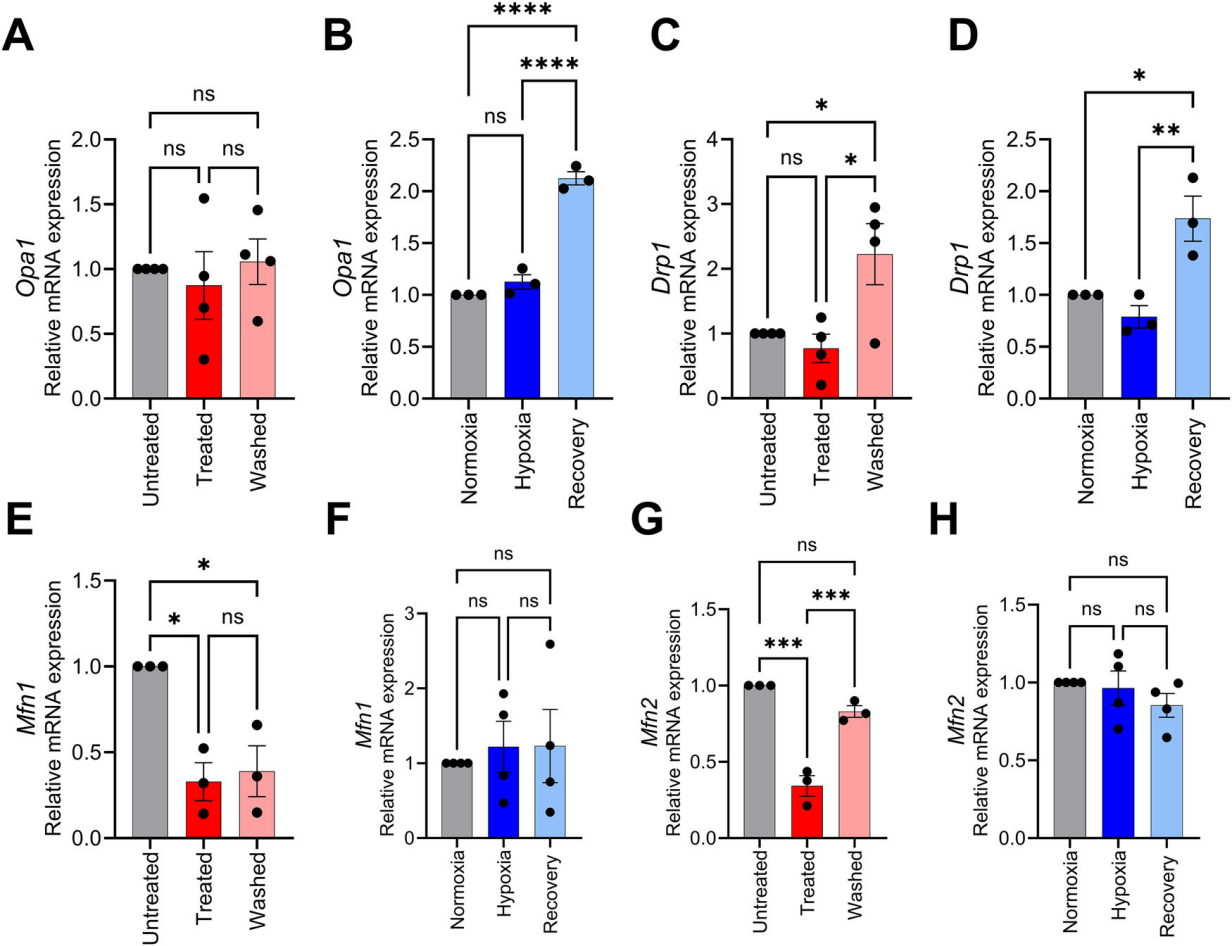
Regulation of mitochondrial fission and fusion during recovery of 661 W cells following apoptotic stimuli induced by staurosporine and hypoxia. Relative mRNA expression of *Opa1* in cells subjected to the **A** staurosporine (*n* = 4) and **B** hypoxia (*n* = 3) protocols. Relative mRNA expression of *Drp1* in cells subjected to the **C** staurosporine (*n* = 4) and **D** hypoxia (*n* = 3) protocols. Relative mRNA expression of *Mfn1* in cells subjected to the **E** staurosporine (*n* = 3) and **F** hypoxia (*n* = 4) protocols. Relative mRNA expression of *Mfn2* in cells subjected to the **G** staurosporine (*n* = 3) and **H** hypoxia (*n* = 4) protocols. Statistical analysis was performed using one-way ANOVA with repeated measures, followed by Tukey’s post hoc test. Significance levels are indicated as * for *p* < 0.05, ** for *p* < 0.01, *** for *p* < 0.001, and **** for *p* < 0.0001.

Mitochondrial fusion and fission are regulators of mitochondrial function, quality control and cellular survival [51]. Therefore, we sought to evaluate the involvement of these processes in recovery from apoptotic insult by evaluating regulators of mitochondrial fusion (MFN1, MFN2, OPA1) and fission (DRP1)[51]. No significant changes were observed in the mRNA levels of *Opa1* in the STR protocol (Fig. 4A). However, a significant increase in mRNA levels of *Opa1* in the recovery phase of the HYP protocol compared both to the Normoxia (*P* = 0.0010) and Hypoxia controls (*P* = 0.0024), as shown in Fig. 4B. Significant increases in mRNA levels of *Drp1* (*DNM1L)*, that codes for Drp1, the primary protein that executes mitochondrial fission [52], were observed in the recovery phase of both the STR and HYP protocols (*P* = 0.0187, *P* = 0.0073, respectively), as shown in Fig.4C,D. Significant reduction in mRNA levels of *Mfn1* was observed for the Treated (*P* = 0.0102) and Washed (*P* = 0.0158) groups compared to the untreated control in the STR protocol, Fig.4E. No changes, however, were observed in *Mfn1* mRNA levels between groups in HYP protocol, Fig.4F. Levels of *Mfn2* mRNA were significantly reduced by STR treatment (*P* = 0.0001, Fig.4G), and significantly increased after removal of STR, reaching baseline levels in the Washed group. No significant changes, however, were observed in the mRNA levels of *Mfn2* in the HYP protocol (Fig. 4H).

### Mitophagy regulation of photoreceptor recovery from apoptotic insult

Mitophagy selectively removes damaged mitochondria, preventing the accumulation of dysfunctional mitochondria that could promote further cellular damage [53]. To assess the role of mitophagy in cellular recovery from apoptotic insult, we evaluated mRNA levels of PTEN induced putative kinase 1 (*Pink1*), FUN14 Domain Containing 1 (*Fundc1*), *Parkin*, and autophagy-related 5 (*Atg5*) and *Map1lc3B*, which encodes the protein microtubule-associated protein 1 light chain 3 beta (LC3B).

**Fig. 5:**
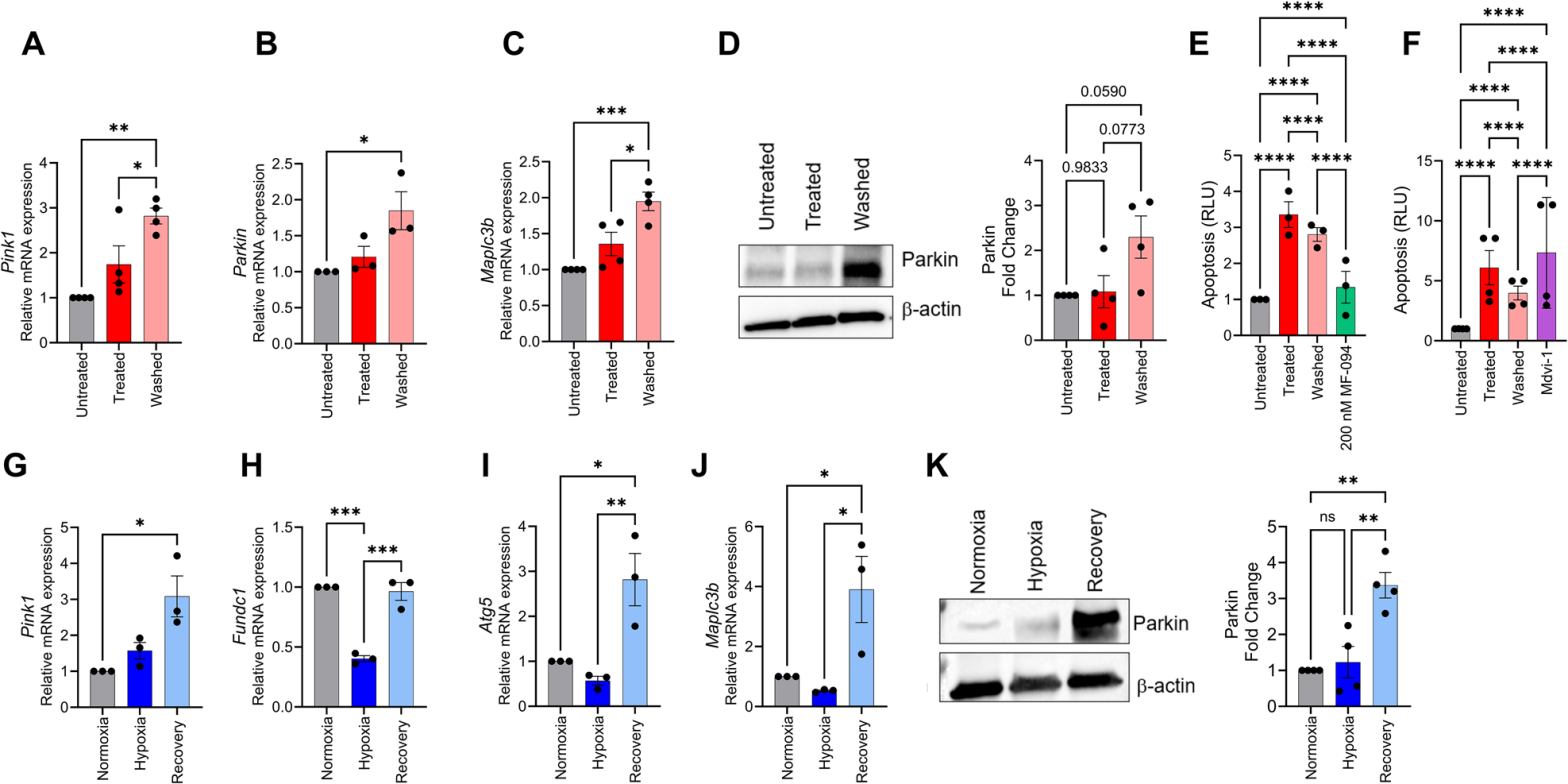
Mitophagy as a key regulator of cellular recovery during cellular recovery from apoptosis. Relative mRNA expression of **A** *Pink1* (*n* = 4), **B** *Parkin* (*n* = 3), and **C** *Maplc3b* (*n* = 4) in cells undergoing the staurosporine protocol. **D** Representative immunoblot for Parkin protein levels, normalized to β-actin in cells subjected to the staurosporine protocol, and respective quantification. Apoptosis (Annexin V) evaluation of untreated, treated, washed groups exposed to the staurosporine protocol, with **E** induction of mitophagy with MF-094 (*n* = 3) and **F** inhibition of mitophagy with Mdivi-1 (*n* = 3). Quantification of relative mRNA expression of **G** *Pink1* (*n* = 3), **H** *Fundc1* (*n* = 3), **I** *Atg5* (*n* = 3), **J** *Maplc3b* (*n* = 3) in cells subjected to the hypoxia protocol. **K** Representatives immunoblot for Parkin protein levels, normalized to β-actin in cells treated under the hypoxia protocol and respective quantification. Statistical analysis **A–D, G–K** was performed using one-way ANOVA with repeated measures, followed by Tukey’s post hoc test. Statistical analysis **E, F** was performed using two-way ANOVA. Significance levels are indicated as * for *p* < 0.05, ** for *p* < 0.01, *** for *p* < 0.001, and **** for *p* < 0.0001.

We started by assessing mitophagy in the STR protocol. Significant increases in mRNA levels of *Pink1* (*P* = 0.0020), *Parkin* (*P* = 0.0316) and *Map1lc3B* (*P* = 0.0009) were observed in the Washed group, compared to the Untreated group (Fig. 5A–C). Parkin protein levels were remarkably increased (Fig. 5D). Given these findings, we hypothesized that mitophagy is central to recovery from apoptotic insult recovery and thus, mitophagy induction would enhance recovery and mitophagy inhibition would attenuate it. To test this hypothesis, we used a mitophagy inducer (MF-094) and inhibitor (Mdivi-1) to assess cellular recovery. Working concentrations were established based on the MTT cell viability/cytotoxicity assay and prior studies in cell culture [54–58], as shown in Supplemental Fig. 3A,B. Working concentrations of 200 nM of MF-094 and 25 µM of Mdivi-1 were selected, and mitophagy induction and inhibition were confirmed in these concentrations Supplemental Fig. 3C. Following the 15 h of STR treatment, media was replaced, and cells were treated with either 200 nM of MF-094 or 25 µM of Mdivi-1, as illustrated in Supplemental Fig. 3D. Next, we evaluated the effects of modulation on apoptosis using Annexin V staining. We found a significant reduction in apoptosis levels in the Washed group compared to the Treated group (*P* < 0.0001), which was further improved by a remarkable prevention of cellular apoptosis with the induction of mitophagy compared to the Treated group and Washed groups (*P* < 0.0001 for both), (Fig. 5E). In contrast, mitophagy inhibition completely blocked cellular recovery from apoptosis (Fig. 5F).

Additionally, we assessed mitophagy in the HYP protocol. We observed a significant increase of *Pink1* mRNA levels in the Recovery group compared to the normoxic control (*P* = 0.0141, Fig.5G). Fundc1 expression was similarly decreased following hypoxia treatment (*P* = 0.0002, Fig. 5H) but returned to baseline levels after 24 h of recovery. Similarly, *Atg5* and *Map1lc3B* were significantly increased in the Recovery group compared to both the Normoxia and Hypoxia groups (Fig.5I, J). Notably, we observed remarkable increases in the levels of Parkin in the group recovering from hypoxic exposure (Fig.5K), which was consistent with the STR protocol.

### Distinct recovery signatures in response to varying stress stimuli

Sun et al. 2017 performed whole-transcriptome RNA sequencing of HeLa cells undergoing cellular anastasis and identified early and late apoptotic profiles [26]. Based on this profile, we examined the early apoptosis genes *Egr1*, *Fos*, *Jun*, *Ier5*, *Ppp1r15a*, and *Ptgs2*, which are known to be rapidly induced in response to cellular stress and play key roles in initiating apoptotic signaling. We also evaluated genes associated with late apoptosis, including *Gadd45* and *Snai1*, which are involved in DNA damage repair, cell cycle arrest, and cellular transitions during the later stages of apoptosis or recovery; and *Efnb2*, a gene implicated in cell signaling and tissue remodeling due its role in the later stages of apoptosis or potential involvement in recovery processes [26]. As shown in Supplemental Fig. 4A–I, we observed that in the HYP protocol, hypoxia treatment induced mRNA expression of *Efnb2* and *Fos* which returned to baseline levels after recovery. Additionally, we observed a significant decrease in mRNA levels of *Fadd45*, *Ppp1r15a, Ptgs2* and *Snai1* in both the hypoxia and recovery groups. *Ier5*, on the other hand, was significantly increased in the Recovery group, compared to both the Normoxia and Hypoxia groups. We found no significant differences in mRNA levels of *Egr1* and *Jun*. Additionally, we evaluated these genes in the STR protocol. As shown in Supplemental Fig. 4J–P, we found that STR treatment induced mRNA expression of *Efnb2*, was significantly decreased in the Washed group in comparison to the Untreated and Treated groups. mRNA levels of *Fos*, *Ier5*, *Jun* and *Ptgs2* were induced by STR treatment, and further induced in the Washed groups. In contrast, mRNA levels of Gadd45 were decreased in both the Treated and Washed groups, compared to the Untreated group. We found no significant differences in mRNA levels of *Egr1*.

### In vivo model of recovery from apoptotic insult and cellular recovery following hypoxic insult

**Fig. 6:**
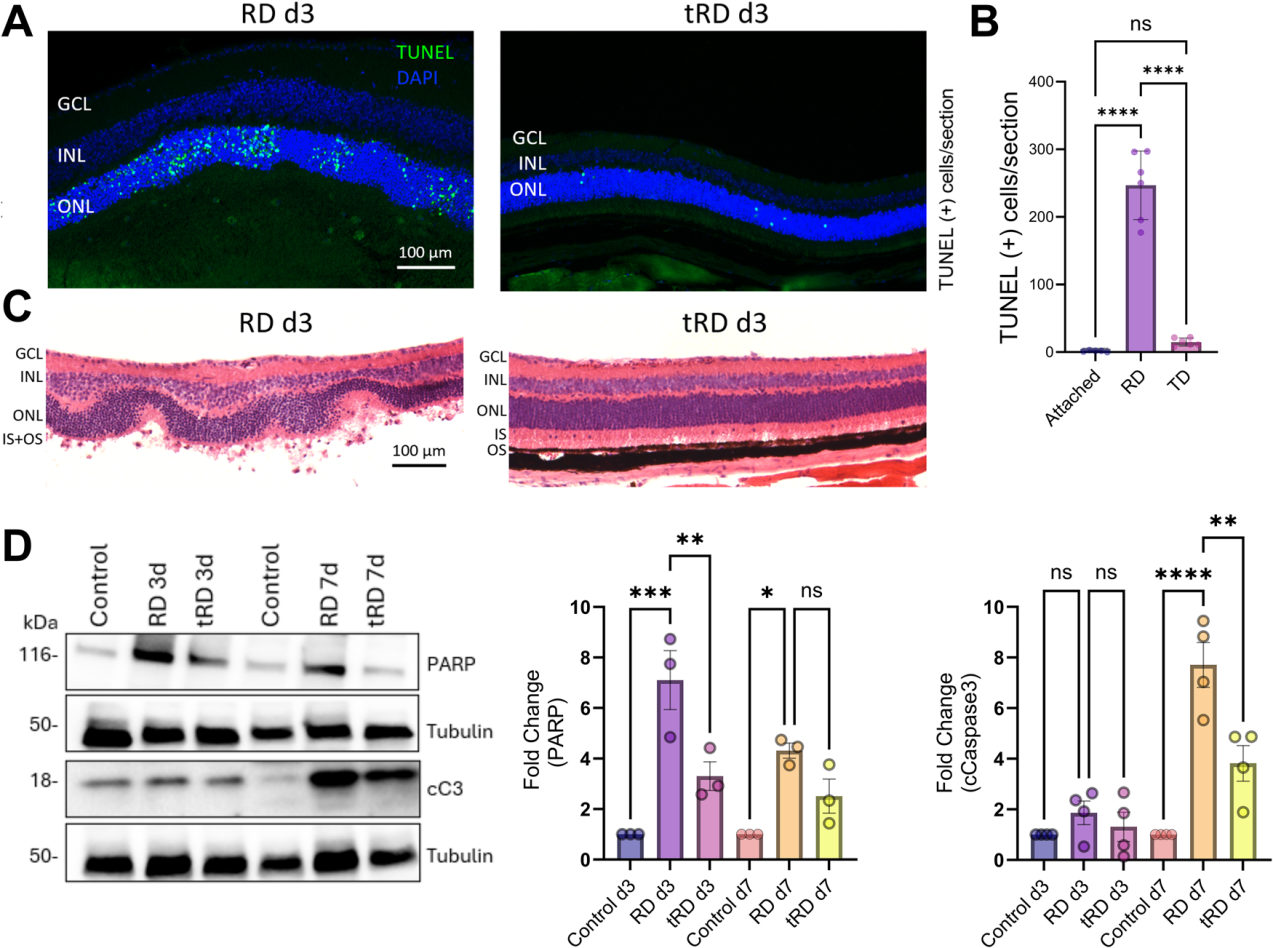
Reduced photoreceptor death and preserved morphology in transient retinal detachment. **A** Representative TUNEL staining on retinal section at 3 days post detachment showing less TUNEL (+) cells in the photoreceptor layer in tRD eye as compared to traditional persistent RD. **B** The respective quantification normalizes the number of TUNEL-positive cells to the thickness of the outer nuclear layer (ONL), (*n* = 6 mice). **C** Representative HE images showing improved morphology of cell layers in the reattached retinas in tRD. **D** Representative immunoblots for apoptosis markers PARP (n = 3), and cleaved caspase 3 (cC3) (*n* = 4), normalized to Tubulin, with respective quantifications for three (3 d) and seven (7 d) days post transient and permanent retinal detachments. Statistical analysis was performed using one-way ANOVA with repeated measures, followed by Tukey’s post hoc test. Significance levels are indicated as * for *p* < 0.05, ** for *p* < 0.01, *** for *p* < 0.001, and **** for *p* < 0.0001, ns: not significant (*p* > 0.05). GCL, ganglion cell layer; INL, inner nuclear layer, ONL outer nuclear layer, IS inner segment, OS outer segment.

We detail in Supplemental Material 1 a simple and reproducible protocol for inducing transient RD (tRD) in mouse using 0.033% sodium hyaluronate to detach approximately 50% of the retina. This protocol resulted in a natural re-attachment of the retina within 3 days, as shown in Supplemental Figs. 5 and 6. Reattached retinas showed significantly fewer TUNEL (+) photoreceptors, as compared to the permanently detached retinas induced with a subretinal injection of 1% sodium hyaluronate (Fig. 6A). There were also fewer immune cells in the subretinal space, as shown in Supplemental Fig. 7A. Both hematoxylin and eosin (HE) (Fig. 6B) and immunohistochemistry (IHC) staining of the photoreceptor proteins rhodopsin and cone opsin (Supplemental Fig. 7B) demonstrate better morphology of inner and outer segments in the reattached retinas. As shown in Fig. 6C, we evaluated the effects of tRD on additional markers of cellular death, such as caspase-3 cleavage, which peaks at 7 days and PARP, which peaks at 3 days. We observed that tRD resulted in significantly less PR cell death.

## Discussion

Here, we demonstrate for the first time in 661 W cells that morphologic apoptotic features such as cytoplasmic shrinkage, cell rounding, and blebbing; as well as functional alterations such as caspase-3 activation, PARP cleavage and phosphatidylserine (PS) externalization can be reversed within 24 h of the removal of the apoptotic stimuli (STR or HYP). Similar recovery from apoptosis has been described in other cell types, such as in the pioneer studies by Tang and collaborators, in which the authors have elegantly demonstrated that ethanol and jasplakinolide-treated HeLa cells could recover and regain functionality after the apoptotic stimuli were removed, and that cells showing typical apoptotic features could recover from morphologic alterations such as cytoplasmic shrinkage, as well as functional perturbations, such as caspase activation, mitochondrial activity [22, 30, 59]. Another very recent study demonstrated that many cell types can recover from death like conditions induced by sublethal L-leucyl-L-leucine methyl ester exposure, as well as by lysosomotropic agents like sphingosine and glycyl-L-phenylalanine 2-naphthylamide. They also found that cells can recover after plasma membrane damage but not after nuclear membrane disruption [60]. While this phenomenon has been previously described before in other cell types, the recovery from apoptosis is particularly important for PR cells, as they are post-mitotic and do not proliferate after reaching maturity [61], meaning that damage or loss of these cells in the retina is irreversible. PR cell death is a leading cause of vision loss in numerous disorders such as RD, retinitis pigmentosa, and age-related macular degeneration, thus the phenomenon described here has important potential for vision preservation [2].

Altogether, our findings suggest a coordinated network of mitochondrial recovery, including the reestablishment of intracellular ATP levels, reduction of mitochondrial ROS, modulation of mitochondrial biogenesis, and mitochondrial fusion/fission, and the degradation of damaged mitochondria (mitophagy) during the recovery phase. Such mitochondrial dynamics are essential for maintaining mitochondrial integrity and function and cellular recovery [62]. Our findings on the central role of mitochondrial function to cellular recovery from apoptosis align with those of other groups [27, 30]. Here, we observed that STR treatment caused a significant decrease in ATP levels, which were restored once the apoptotic stimulus was removed. This finding suggests a restoration of mitochondrial function in the recovery phase. This has been described before in RGCs where the studies have shown that RGCs exhibiting significant mitochondrial fragmentation and a loss of membrane potential are capable of reversing these damages, highlighting an intrinsic window of cellular resilience and the potential for therapeutic intervention to preserve retinal function [20]. In contrast, HYP treatment did not significantly affect intracellular ATP levels during the stress phase but induced a two-fold increase in these levels in the recovery phase, which recapitulated the proliferation results. Together, these findings indicate that 661 W cells regain their homeostatic proliferative capacity within 24 h of recovery in the HYP protocol (24-h doubling time). These findings are supported by those of Tang and collaborators and indicate functional recovery of cellular proliferation [59]. However, proliferative capacity was not restored within 24 h in the STR group, though further time points were not tested. Notably, flow cytometry results from the STR protocol at the 24-h time point revealed a remarkable reduction in the proportion of both early and late apoptotic cells. Although the overall cell number remained similar, as indicated by the crystal violet assay, which stains cellular components such as DNA and proteins and serves as a normalization method for adherent cells [63], the proportion of healthy cells increased substantially following STR removal. Specifically, the percentage of healthy cells increased from only 14% immediately after STR treatment to 51.7% after 24 h of recovery. Along with morphological data, these findings suggest that the observed recovery is not due to the survival of a subpopulation of less damaged cells, nor the proliferation of new cells, but rather reflects recovery of cells from apoptosis, consistent with recovery from apoptotic insult. Nonetheless, an important limitation of this study is that our experiments do not yet provide single-cell evidence for the reversal of apoptotic markers, which would be required to establish that photoreceptor cells have undergone anastasis. Future studies will focus on developing methodologies for photoreceptor cells.

The removal of the apoptotic-inducing stressor caused a significant reduction in mitochondrial ROS levels in both protocols. These findings agree with previous reports connecting mitochondrial ROS to the regulation of apoptosis and survival [64, 65]. Moreover, the significant increase in mitochondrial transcript ratio in the recovery phases of both protocols further indicate recovery in mitochondrial function [66]. Our study also reveals that there is a distinct role for mitochondrial biogenesis during STR and HYP stresses. While STR-treatment increased mRNA of genes involved in mitochondrial biogenesis during the stress phase, possibly as an attempt to maintain intracellular ATP levels, HYP-treatment increased these markers in the recovery phase, similar to what was described in recovering neuronal PC12 cells [27]. Also, recently a research study showed that activation of the soluble adenylyl cyclase (sAC)/cAMP/PKA pathway in mouse RGCs under oxidative stress promoted mitochondrial biogenesis, preserved mitochondrial structure and increased ATP production suggesting that enhancing mitochondrial biogenesis can protect RGCs in conditions such as glaucoma [20]. Altogether, these findings highlight how mitochondrial dynamics are tightly regulated under different stress conditions.

Mitochondrial fission/fusion plays a major role in maintaining mitochondrial quality control and function following stress [51, 67]. The findings in our study suggest that STR and HYP treatments modulate fusion and fission mRNA levels, reinforcing the idea that maintaining mitochondrial dynamics is essential for cellular homeostasis and stress adaptation. The balance between these processes may help to prevent mitochondrial dysfunction, which is often associated with various diseases [51, 68]. Mitochondrial fusion and fission are crucial processes that maintain mitochondrial health. Fusion helps repair damaged mitochondria by merging them and sharing contents, while fission divides mitochondria, enabling the removal of damaged ones through mitophagy. Mitophagy, the selective autophagy of damaged mitochondria, is critical for promoting mitochondrial homeostasis and cell survival in conditions of stress [69]. Increasing evidence [70–74] has indicated that controlled enhancement of mitophagy is a promising therapeutic strategy in the management of neurodegenerative diseases, as recently reviewed [75]. A previous study from our group demonstrated that 661 W cells showed early activation of autophagy following RD or Fas-receptor activation, with Atg5 and LC3BII peaking at 3 days, followed by a decline as Calpain 1 activity increased at day-7. Calpain inhibition enhanced autophagy, reduced caspase-8 activation, and decreased PR apoptosis, promoting cell survival suggesting that retinal cells can undergo recovery from near cell death state when pro-survival pathways like autophagy are supported [38]. In this study, we observed a significant increase in markers associated with mitophagy and autophagy [76], including mRNA levels of *Pink1*, *Fundc1*, *Atg5*, and *Maplc3b* and protein levels of Parkin during the recovery phase after both STR and HYP treatments, supporting the involvement of mitophagy in the recovery of photoreceptor cells after apoptotic stress. We thus hypothesized that mitophagy had a causal role in 661 W cellular recovery after apoptotic stress. To test this, we evaluated the effect of a selective mitophagy inducer, MF094, a USP30-specific inhibitor. USP30 inhibition prevents the removal of ubiquitin from outer mitochondrial membrane proteins, which stabilizes PINK1 and Parkin activity, promoting ubiquitin signaling and facilitating autophagic receptor recruitment [57, 75]. We found that selective induction of mitophagy significantly inhibited apoptosis in 661 W cells. In contrast, treatment with Mdivi-1, which inhibits mitophagy by interfering with mitochondrial fission, a crucial step in mitophagy [77], caused a significant increase in apoptosis, suggesting a central role of mitophagy in cellular recovery from apoptotic insult. A limitation of this study is that the current findings demonstrate an association rather than direct causality between mitophagy and recovery from apoptosis. Genetic approaches will be employed in the future to establish a causal relationship.

The analysis of anastasis-related genes in STR and HYP treatments reveals distinct expression patterns tied to apoptosis and recovery. In the HYP protocol, early apoptotic genes (e.g., *Fos*, *Efnb2*) were elevated during hypoxia and returned to baseline during recovery, suggesting an initial stress response followed by a recovery phase. Meanwhile, late apoptotic genes (e.g., *Gadd45*, *Ppp1r15a*, *Snai1*) were decreased during recovery, indicating a shift away from cell death. In the STR protocol, genes such as *Fos*, *Ier5*, and *Ptgs2* were induced during treatment and further increased during recovery, suggesting a sustained pro-apoptotic or stress response. In contrast, *Gadd45* was decreased, possibly reflecting a transition to cell survival or repair. These patterns suggest that the two treatments modulate apoptotic and recovery pathways differently, with varying impacts on stress response, apoptosis, and cellular recovery.

Clinical studies have suggested that when the detached retina is reattached surgically within about 1 week after RD, many macula-involving RD patients can recover close to their baseline visual acuity [78–80]. Visual recovery significantly decreases if the reattachment occurs after 7 days of detachment, however, patients still typically recover significant vision compared to before RD repair [80]. These findings suggest that PR cells can recover from apoptotic stress if it is removed before the point of no-return. A major gap in knowledge had been the molecular events that occur within PR cells upon reattachment of the retina to the RPE. In part, this deficit results from a limitation in the current animal models of RD. The majority of the current models of experimental RD create the retina-RPE separation by subretinal injection of 1% sodium hyaluronate [13, 18, 81–84]. In these models, the retina remains detached indefinitely, with minimal resolution of the detachment. Thus, to test our hypothesis that PR can recover in vivo, we developed a simple and reproducible protocol for inducing transient RD (tRD) in mouse using 0.033% sodium hyaluronate to detach approximately 50% of the retina. This protocol results in a natural re-attachment of the retina within 3 days. We found that, similar to our in vitro model of cellular recovery, removing the stressor, which in this case is hypoxia induced by the physical separation between the retina and the RPE, can also promote cellular recovery in vivo. Furthermore, we propose that this model can serve as a valuable tool for studying mechanisms involved in the recovery process of photoreceptors following separation from the RPE, such as occurring in RD.

## Conclusion

Our study demonstrates that apoptotic morphological and functional alterations can be reversed in 661 W photoreceptor cells within 24 h of apoptotic stimulus removal. We identified a mitochondrial recovery signature involving ATP restoration, reduction of mitochondrial ROS, regulation of mitochondrial biogenesis, and modulation of fusion, fission, and mitophagy, with distinct mitochondrial adaptations to stress in STR- and HYP-induced cells. In addition, we found a central role for mitophagy in 661 W cell survival, as its selective induction reduced apoptosis, while its inhibition exacerbated cell death. Importantly, we validated the in vitro findings of our cellular model in a novel animal model, indicating a role for recovery from apoptotic insult in photoreceptor cells also in vivo. Given the therapeutic potential of mitophagy modulation in neurodegenerative diseases, targeting mitochondrial quality control may offer a promising strategy for protecting photoreceptor function and preventing vision loss and should be further explored in future research.

## Supplementary information

Supplementary Video 1

Supplemental Material

Dataset 1

## Data Availability

All data generated and analyzed in this study are presented in this published article. Primary data may be made available from the corresponding author upon reasonable request.
