# Supplementary material for: Recovery from apoptosis in photoreceptor cells: A role for mitophagy
Source: Cell Death Dis. 2026 Jan 30;17(1):167. doi: 10.1038/s41419-026-08436-3 (PMC12877012; doi:10.1038/s41419-026-08436-3)
Supplement: Supplementary file 2 — Supplemental Material [file 41419_2026_8436_MOESM2_ESM.docx]

**Supplemental Material**

***Recovery from Apoptosis in Photoreceptor Cells: A Role for Mitophagy***

**Transient Detachment Protocol**

1. **MATERIALS AND SUPPLIES**
   1. **Reagents**

- 1% Sodium hyaluronate: 10 mg/mL, Healon OVD (Abbott Medical Optics, Uppsala, Sweden)
- Ultra-pure PBS (Gibco,10010-023)
- Ketamine 100 mg/ml (Covetrus, North America LLC, NDC# 11695070301)
- Xylazine (VET ONE, AnaSed LA 100 mg/mL, NDC 13985-704-10)
- Tropi-Phen eye drop (1% tropicamide, 2.5% phenylephrine; PINE Pharmaceuticals)
- Lubricant eye gel (GenTeal Tears, 10 g; Alcon)
- Lubricant eye drop (Systane, Alcon 30 mL, Alcon)

- 1. **Equipment**
- 3 mm cover slides (Warner Instruments, 64-0720)
- 5 μL Gastight Hamilton syringe (Hamilton, 80016)
- Beveled 34-gauge needle (Hamilton 207434, at 0.5-inch length with needle point style 4 at 30 degrees)
- 300 μL insulin syringe with 31-gauge needle (UltiCare VetRx, 09436)
- Gel heating pads (Braintree Scientific, 39DP)
- Surgical microscope (Zeiss, SN 6137102794, REF# 6137)
- Optical Coherence Tomography (OCT) (Bioptigen)

1. **TRANSIENT RETINAL DETACHMENT PROCEDURE**
   1. **Preparation of 0.033% hyaluronic acid solution (serial dilutions)**
      1. Prepare a 0.5% sodium hyaluronate solution: Transfer 100 μL of 1% sodium hyaluronate (undiluted Healon OVD) and 100 μL PBS into a sterilized microfuge tube. Mix thoroughly.
      2. Prepare a 0.2% sodium hyaluronate solution: Transfer 100 μL of the 0.5% sodium hyaluronate solution and 150 μL PBS into a sterilized centrifuge tube. Mix thoroughly.
      3. Prepare a 0.1% sodium hyaluronate solution: Transfer 100 μL of the 0.2% sodium hyaluronate solution and 100 μL PBS into a sterilized centrifuge tube. Mix thoroughly.
      4. Prepare a 0.1% sodium hyaluronate solution: Transfer 100 μL of the 0.2% sodium hyaluronate solution and 100 μL of PBS into a sterilized centrifuge tube. Mix thoroughly.
      5. Prepare a 0.033% sodium hyaluronate solution: Transfer 100 μL of the 0.1% Hyaluronic acid solution and 200 μL PBS into a sterilized centrifuge tube. Mix thoroughly. Store at 4 °C.
   2. **Preparation of mice and anesthesia**
      1. Dilate pupil with topical Tropi-Phen eye drop.
      2. Anesthesia: intra-peritoneal injection of premixed cocktail of ketamine at 80 mg/kg and xylazine at 10 mg/kg.
      3. Prepare a heating pad for recovery after surgery to avoid hypothermia.
   3. **Retinal Detachment**
      1. Place mouse under the surgical microscope with left eye facing up and being perpendicular to the microscope light. Adjust the focus, intensity of the light, and magnification.
      2. Load the Hamilton syringe with 3 mL of 0.033% sodium hyaluronate and attach needle.
      3. Elevate and stabilize the head of the mouse on a head rest made with folded tissue.
      4. Create a sclerotomy 1 mm posterior to the limbus at the superior aspect of the eye (12 o’clock position) using the tip of a 30.5-gauge needle attached to a 300 μL insulin syringe, carefully avoiding lens damage.
      5. Under the microscope, apply a small amount of Healon OVD on the cornea, and place a 3mm cover slide to visualize the retina.
      6. Insert the 34-gauge needle through the sclerotomy into the vitreous cavity at a 60-degree angle to avoid the lens.
      7. While inserting the needle inside the vitreous cavity and using direct visualization through the microscope, tilt the syringe to make the needle move towards the nasal part of the peripheral retina, with bevel facing the retina.
      8. Select an area that is away from the retinal vessels to avoid hemorrhage.
      9. Create a retinotomy by gently pushing the beveled needle into the subretinal space.
      10. Then, slowly inject the 0.033% sodium hyaluronate into the subretinal space to create the retinal detachment. In each experimental eye, 3 μL will detach approximately half of the retina, primarily on the nasal side. The temporal half of the retina remains attached as control.
      11. Slowly withdraw the needle avoiding touching the lens.
      12. Apply lubricating eye gel to the eye while the animal recovers from anesthesia on the heating pad.
   4. **Post procedure assessment**
      1. Optical Coherence Tomography

Optical coherence tomography (OCT) was performed using the spectral domain OCT equipment provided by Bioptigen, Inc. (Durham, NC, USA). Volume scan was set at 1.6 x 1.6 mm square. Perform 2 scans, one with the optic nerve positioned at one side edge of the scan field to scan the detached half of the retina, then the second scan with the optic nerve positioned at the opposite edge of the field to scan the attached half of the retina. Perform OCT exam right after RD and at 1-, 2-, 3-, 5-, and 7 days post RD.

- - 1. Immunofluorescence on Retinal Sections

Retinal sections crossing the optic nerve were used for staining. RHO and m-Opsin staining was performed on 10 µm sections, whereas Iba1 staining utilized 30 µm sections. After blocking with 5% goat serum in 0.1% Triton X-100 in PBS (PBST) for an hour, and raising for three times with PBST, sections were incubated overnight at 4°C with primary antibodies. After washes with PBST and incubated with secondary antibodies for 1 hour at room temperature, slides were then mounted using ProLong Gold with DAPI. Images were taken at the comparable retina area (600 µm from the optic nerve) using a confocal microscope at a fixed gain.

1. **POTENTIAL PITFALLS AND TROUBLE SHOOTING**

We find that the position of the left eye is more natural and easier for retinal detachment. When detaching the retina of the left eye of the mouse, the sclerotomy will be create at the superior aspect of the eye (around 12 o’clock position), and the nasal part of the retina will be detached. If the detachment needs to be performed to the retina of the right eye, we suggest creating the sclerotomy at the inferior aspect of the eye (around 6 o’clock position).

In our previous work, we used 0.5% tropicamide and 2.5% phenylephrine separately to dilate the pupils and we often found that pupils were dilated slow and not large enough. We recently switched to a cocktail eye drop, Tropi-Phen eye drop, a mixture of 1% tropicamide and 2.5% phenylephrine (PINE Pharmaceuticals), which provides more rapid and prolonged dilation.

When making the sclerotomy, if bleeding occurs due to the rupture of the blood vessel around the limbus, gently wipe off the blood and press the wound with a sterilized swab. To avoid vitreous hemorrhage caused by the injury to retinal blood vessel, it is essential to avoid the retinal vessel and choose a peripheral area away from the retinal vasculature to create a retinotomy. When a retinotomy is not created properly and the needle is not advanced into the subretinal apace, sodium hyaluronate can be delivered in the vitreous cavity instead of the subretinal space. We found that it is critical to keep a clear visualization of the needle tip when touching the retina. The operator can adjust the focus of the microscope to optimize the visualization to ensure a successful retinotomy. We also suggest avoiding excessive force when creating a retinotomy. A gentle pressure is sufficient to advance the needle into the subretinal space. Excessive pressure can also result in damage to the underlying RPE and even the bleeding from the choroid.

In our protocol, injection of 3 μL of 0.033% Sodium hyaluronate into the subretinal space will detach approximately half of the retina, primarily on the nasal side. The area of detached retina should be easily visualized under the microscope after surgery; however, performing OCT scans can also help assess the success of the retinal detachment. Follow-up OCT scans at 1-, 3-, 5- and 7- days post-procedure can help with monitoring the reattachment of the retina. A drawback of frequent OCT is extra anesthesia to the animal.

**Supplemental Figures**

**Supplemental Figure 1. Flow cytometry evaluation of Annexin/PI staining of 661W cells in the staurosporine protocol**.

**A** Group without staurosporine treatment (Untreated); **B** Group receiving 0.05 µM staurosporine (STR) for 15 hours (Treated); **C** Group receiving 0.05 µM staurosporine for 15 hours, followed by 24 hours of recovery (Washed). The Q1 quadrants represent early apoptotic cells (Annexin V⁺/PI⁻); the Q2 quadrants represent late apoptotic cells (Annexin V⁺/PI⁺); the Q3 quadrants represent necrotic cells (Annexin V⁻/PI⁺) and the Q4 quadrants represent healthy/viable cells (Annexin V⁻/PI⁻). **D** Comparative graphs showing the quantitative analysis based on staining profiles.

**Supplemental Figure 2. Effect of hypoxia and staurosporine anastasis protocol on 661W cellular proliferation.**

**A** Cellular proliferation assessed by the crystal violet methodology after 24 hours in the Staurosporine protocol. **B** Cellular proliferation assessed by the crystal violet methodology after 24 hours in the Hypoxia protocol. **C** Cellular proliferation assessed by the crystal violet methodology after 12 hours of recovery in the Hypoxia protocol. **D** Apoptosis (Annexin V) evaluation of untreated, treated, washed groups exposed to 12 hours of recovery in the Hypoxia protocol. **E** Immunoblots showing levels of cleaved caspase 3 (cC3), normalized to Tubulin, after 12 h of recovery in the Hypoxia protocol, and respective quantifications, and respective quantification. Data are presented as mean ± SEM. Statistical significance was assessed using one-way ANOVA with repeated measures, followed by Tukey's post hoc test. Significance levels are indicated as *p < 0.05, **p < 0.001, ***p < 0.001, ****p < 0.0001 and ns: non-significant. FC: fold change.

**Supplemental Figure 3. Establishing effective concentrations of the mitophagy inducer (MF-094) and inhibitor (Mdivi-1).**

The MTT assay was used to evaluate cell viability at varying concentrations of **A** MF-094 (20–200 nM), and **B** Mdivi-1 (0.5–750 µM). **C** Immunoblots showing levels of mitophagy marker Parkin, normalized to Tubulin, upon 24 hours of treatment with mitophagy inhibitor (Mdivi-1) and inducer (MF-094). **D** Schematic representation of mitophagy induction and inhibition to promote cellular anastasis in 661W photoreceptor cells. Data are presented as mean ± SEM. Statistical significance was assessed using one-way ANOVA with repeated measures, followed by Tukey's post hoc test. Significance levels are indicated as ***p < 0.001 and ****p < 0.0001.

**Supplemental Figure 4. Effect of hypoxia and staurosporine anastasis protocol on anastasis signature genes**

Quantification of relative mRNA expression of **A** *Efnb2*, **B** *Egr1*, **C** *Fos*, **D** *Gadd45,* **E** *Ier5*, **F** *Jun*, **G** *Ppp1r15a*, **H** *Ptgs2, and* **I** *Snai1* in cells subjected to the hypoxia anastasis protocol.

Quantification of relative mRNA expressions of **J** *Efnb2*, **K** *Egr1*, **L** *Fos*, **M** *Gadd45,* **N** *Ier5*, **O** *Jun, and* **P** *Ptgs2* in cells subjected to the staurosporine anastasis protocol.

Statistical analysis was performed using one-way ANOVA with repeated measures, followed by Tukey's post hoc test. Significance levels are indicated as * for p < 0.05, ** for p < 0.01, *** for p < 0.001, and **** for p < 0.0001.

**Supplemental Figure 5. Representative images of a successful transient retinal detachment (tRD).**

Representative photos showing **A** retinal detachment of the nasal part of the retina immediately after, day zero (d0) subretinal injection of 3 μL of 0.033% Sodium hyaluronate and **B** reattached retina at 3 days (d3) after induction of the tRD. Red arrows indicate the site of retinotomy. T: temporal; N: nasal.

**Supplemental Figure 6.** **Representative OCT images of a successful tRD.**

Representative OCT scan photos showing **A** persistent detachment of nasal side of the retina in traditional RD at day 0 (immediately after subretinal injection), day1 and day3 post RD; while in tRD eye, detached retina is reattached at 3 days post tRD. **B** Representative paired OCT scan and fundus image showing reattached retina when scanning cross the optic nerve, and red arrows indicate the site of the retinotomy in a scan image crossing the injection site.

**Supplemental Figure 7.** **Preserved morphology and reduced photoreceptor activation of the immune cells in tRD.**

**A** Representative images of Iba1 staining on retinal section at 5 days post detachment showing fewer Iba1 (+) cells in the ONL and subretinal space in tRD eye. **B** Representative IHC staining RHO (rhodopsin) and m-opsin at 3 days post detachment, showing improved morphology of inner and outer segments in the reattached retinas.

GCL, ganglion cell layer; INL, inner nuclear layer, ONL, outer nuclear layer; IS, inner segment; OS, outer segment.

**Supplemental Figure 8. Schematic of the retinal detachment procedure.**

Retinal detachment (frontal view) on **A** left eye, and **B** right eye.

**Supplemental Tables**

**Supplemental Table 1. Materials and reagents**

| **Reagent** | **Supplier** | **Catalog number** |
| --- | --- | --- |
| Antibodies | | |
| Cleaved caspase 3 | Novus Biologicals | NB100-56113 |
| PARP | Cell Signaling Technology | 46D11 |
| Parkin | Cell Signaling Technology | 4211S |
| Fundc1 | Novus Biologicals | NBP1-81063 |
| β-actin | BD Biosciences | 612656 |
| Tubulin | Sigma | T8203 |
| Iba1 (1:100) | Novus Biologicals | NB100-1028 |
| Rhodopsin (1:2000) | Novus Biologicals | NBP1-48334 |
| M-opsin (1:1000) | Millipore | AB-5405 |
| Secondary antibody (anti-mouse) | Cytiva | NA931V |
| Secondary antibody (anti-rabbit) | Cell Signaling Technology | 7074S |
| Cell culture | | |
| DMEM | Thermo Scientific | 11995-065 |
| Fetal bovine serum | Invitrogen | A5670801 |
| Penicillin–streptomycin | Thermo Scientific | 15070-063 |
| Putrescine | Sigma | P5780 |
| β-Mercaptoethanol | Sigma | 444203 |
| Hydrocortisone 21-hemisuccinate | Sigma | H2270 |
| Progesterone | Sigma | P8783 |
| Transient and Permanent Detachment: reagents and critical material | | |
| 1% Sodium hyaluronate, 10 mg/mL, Healon OVD | Johnson & Johnson Vision | |
| Ultra-pure PBS | Gibco | 10010-023 |
| Ketamine 100mg/mL | Covetrus, North America LLC | NDC# 11695070301 |
| Xylazine 100mg/mL | VET ONE, AnaSed LA | NDC 13985-704-10 |
| Tropi-Phen eye drop - 1% tropicamide, 2.5% phenylephrine | PINE Pharmaceuticals | 2001094 |
| GenTeal Tears - Lubricant eye gel | Alcon | 1001186601 |
| Systane - Lubricant eye drop | Alcon |  |
| 5 μL Gastight Hamilton syringe | Hamilton | 80016 |
| 3 mm cover slides | Warner Instruments | 64-0720 |
| Beveled 34-gauge needle, at 0.5-inch length with needle point style 4 at 30 degrees | Hamilton | 207434 |
| 300 μL insulin syringe with 31-gauge needle | UltiCare VetRx | 09436 |
| Gel heating pads | Braintree Scientific, INC | 39DP |
| Other Chemicals, Reagents, Material | | |
| Modular Incubator Chamber / Hypoxia Chamber | Embrient Inc | MIC-101 |
| Fast SYBR Green Master Mix | Invitrogen | 4385612 |
| Hematoxylin | Fisher Scientific | H345-25 |
| Eosin | Fisher Scientific | E511-25 |
| Paraformaldehyde | Electron Microscopy Sciences | 15710 |
| RIPA | Sigma | R0278 |
| Protease inhibitor | Thermo Scientific | A32955 |
| Phosphatase inhibitor | Thermo Scientific | A32957 |
| ProLong Gold with DAPI | Invitrogen | P36941 |
| NuPAGE LDS Sample Buffer | Thermo Scientific | NP0007 |
| Reducing Agent | Thermo Scientific | NP0009 |
| 4%-15% SDS-PAGE gel  (Mini-PROTEAN TGX) | Bio-Rad | 4561083 |
| Trans-Blot Turbo Transfer Pack | Bio-Rad | 1704156 |
| Bovine serum albumin (BSA) | Sigma | A9647 |
| Tris-buffered saline (TBS) | Bio-Rad | 1706435 |
| Triton™ X-100 | Sigma | T8787 |
| Tween®20 | Sigma | P9416 |
| SuperSignal West Pico PLUS Chemiluminescent Substrate | Thermo Scientific | 34580 |
| Trypsin | Thermo Scientific | 25200-056 |
| Crystal violet | Sigma | C6158 |
| Methanol | Fisher Chemical | A412-4 |
| Thiazolyl Blue Tetrazolium Bromide | Sigma | M5655 |
| Isopropanol | Sigma | I9516 |
| Mitochondrial Division Inhibitor, Mdivi-1 | Sigma | 475856 |
| MF-094 | Sigma | SML2501 |
| DRAQ5 | Thermo Scientific | 62254 |
| MitoSOX | Invitrogen | M36008 |
| DCFDA | Invitrogen | C6827 |
| 35 mm glass-bottom dishes | Ibidi | 81218-200 |
| EDTA disodium salt (EDTA- Na₂) | Thermo Fisher Scientific | S311-500 |
| Sodium chloride (NaCl) | Thermo Fisher Scientific | S271-3 |
| Disodium phosphate (HNa_2_O_4_P) | Thermo Fisher Scientific | BP332-1 |
| Potassium chloride (KCl) | Sigma | P9541 |
| Commercial kits | | |
| RC DC Protein Assay Kit | Bio-Rad | 5000120 |
| RNeasy Plus Mini kit | Qiagen | 74134 |
| High-Capacity cDNA Reverse Transcription Kit | Invitrogen | 4368814 |
| ATPlite Luminescence Assay System | Revvity | 6016943 |
| RealTime-Glo Annexin V Apoptosis Assay | Promega | JA1000 |
| DeadEnd™ Fluorometric TUNEL System | Promega | G3250 |
| Dead Cell Apoptosis Kits with Annexin V for Flow Cytometry (FITC, Propidium Iodide) | Invitrogen | V13242 |
| Critical equipment | | |
| CFX384 | Bio-Rad | |
| c500 chemiluminescence imager | Azure Biosystems | |
| Trans-Blot Turbo Transfer System | Bio-Rad | |
| Surgical microscope | Zeiss SN 6137102794, REF# 6137 | |
| Optical Coherence Tomography (OCT) | Bioptigen, Inc. Durham | |
| Microscope | DM6000, Leica | |
| Confocal Microscope | STELLARIS 8 FALCON, Leica | |
| Microtome | Shandon AS325, Thermo Scientific | |
| Attune NxT Cytometer | Invitrogen, Thermo Fisher Scientific | |
| Software |  | |
| GraphPad Prism | https://www.graphpad.com/ Version 10.2.2 | |
| BioRender | https://biorender.com/ | |
| ImageJ | ImageJ.JS | |

**Supplemental Table 2. Primer sequences used for qRT-PCR analysis**

| **Gene (mouse)** | **Forward sequence** | **Reverse sequence** |
| --- | --- | --- |
| *Atg5* | CTTGCATCAAGTTCAGCTCTTCC | AAGTGAGCCTCAACCGCATCCT |
| *Drp1* | TGGAAAGAGCTCAGTGCTGG | ACTCCATTTTCTTCTCCTGTTGT |
| *Efnb2* | GTGCTCTCCTGACTGCTTAGTG | GAGAGACGAGTCGGTGTGAGG |
| *Egr1* | TTCAATCCTCAAGGGGAGCCG | CGATGTCAGAAAAGGACTCTGTGG |
| *Fos* | GTGAAGACCGTGTCAGGAGG | AGTTGATCTGTCTCCGCTTGG |
| *Fundc1* | AGACACCACTGGTGGAATCGAG | CCTTCTGGAATAAAAATCCTGCAC |
| *Gadd45b* | GCCTCCTGGTCACGAACTGTC | GGGACCCATTGGTTATTGCCT |
| *Ier5* | ACGCGTCACCAGGTCTTTTC | TGTAGGCGGGATCAGGTCTT |
| *Jun* | TGAAGTGGCATGTGCTGTGA | TTCCATGGGTCCCTGCTTTG |
| *Lc3-II* | CAAGCCTTCTTCCTCCTGGTGAA | CCATTGCTGTCCCGAATGTCTCC |
| *Map1lc3b* | GTCCTGGACAAGACCAAGTTCC | CCATTCACCAGGAGGAAGAAGG |
| *Mfn1* | CACTTTTGCTCGACTGTGCC | CTCGGGTGGAGAAACTGCTT |
| *Mfn2* | ACCAGCTAGAAACGAGATGTCC | GTGCTTGAGAGGGGAAGCAT |
| *Mt9/mt11* | GAGCATCTTATCCACGCTTCC | GGTGGTACTCCCGCTGTAAA |
| *Ndufv1* | CTTCCCCACTGGCCTCAAG | CCAAAACCCAGTGATCCAGC |
| *Opa1* | ATTTCGCTCCTGACCTGGAC | GGTGTACCCGCAGTGAAGAA |
| *Paris* | AGTTGGACTCTGGAGCAGGA | GCTGCTGTGTTGAGCTTCAG |
| *Parkin* | AAACCGGATGAGTGGTGAGT | AGCTACCGACGTGTCCTTGT |
| *Pgc1α* | GCAGTCGCAACATGCTCAAG | GGGAACCCTTGGGGTCATTT |
| *Pink1* | GAGCAGACTCCCAGTTCTCG | GTCCCACTCCACAAGGATGT |
| *Ppp1r15a* | CAGAGGCGGCTCAGATTGTT | ATCTCGTGCAAACTGCTCCC |
| *Ptgs2* | AAGCCTTCTCCAACCTCTCCT | GGAAGCTCCTTATTTCCCTTCACA |
| *Pum1* | ACAGCCTGCCAACACGTCCTTG | CCACTGCCAGTGTTGGAGTTTG |
| *Sirt1* | ACCAGTAGCACTAATTCCAAGTTC | TGGCATACTCGCCACCTAAC |
| *Snai1* | AGGACGCGTGTGTGGAGTT | ACTTGGGGTACCAGGAGAGAG |
